# Use of ramucirumab for various treatment lines in real-world practice of patients with advanced hepatocellular carcinoma

**DOI:** 10.1186/s12876-023-02674-x

**Published:** 2023-03-11

**Authors:** Naoya Kanogawa, Sadahisa Ogasawara, Susumu Maruta, Yotaro Iino, Masamichi Obu, Takamasa Ishino, Keita Ogawa, Sae Yumita, Terunao Iwanaga, Hidemi Unozawa, Miyuki Nakagawa, Kisako Fujiwara, Takafumi Sakuma, Naoto Fujita, Ryuta Kojima, Hiroaki Kanzaki, Keisuke Koroki, Kazufumi Kobayashi, Masanori Inoue, Soichiro Kiyono, Masato Nakamura, Takayuki Kondo, Tomoko Saito, Ryo Nakagawa, Shingo Nakamoto, Ryosuke Muroyama, Tetsuhiro Chiba, Ei Itobayashi, Yoshihiro Koma, Ryosaku Azemoto, Jun Kato, Naoya Kato

**Affiliations:** 1 Department of Gastroenterology, Graduate School of Medicine, Chiba University, 1-8-1 Inohana, Chuo-Ku, Chiba, 260-8670 Japan; 2 Department of Gastroenterology, Asahi General Hospital, Asahi, Japan; 3 Department of Gastroenterology, Kimitsu Chuo Hospital, Kisarazu, Japan

**Keywords:** Hepatocellular carcinoma, Ramucirumab, Late-line, REACH-2

## Abstract

**Purpose:**

Ramucirumab was shown to be effective as a second-line treatment after sorafenib in patients with advanced hepatocellular carcinoma (HCC) with alpha-fetoprotein levels > 400 ng/mL in a worldwide phase 3 trial. Ramucirumab is used in patients pretreated with various systemic therapies in clinical practice. We retrospectively examined the treatment outcomes of ramucirumab administered to advanced HCC patients after diverse systemic therapies.

**Methods:**

Data were collected from patients with advanced HCC who received ramucirumab at three institutions in Japan. Radiological assessments were determined according to both Response Evaluation Criteria in Solid Tumours (RECIST) version 1.1 and modified RECIST and the Common Terminology Criteria for Adverse Events version 5.0 was used to assess adverse events.

**Results:**

A total of 37 patients treated with ramucirumab between June 2019 and March 2021 were included in the study. Ramucirumab was administered as second, third, fourth, and fifth-line treatment in 13 (35.1%), 14 (37.8%), eight (21.6%), and two (5.4%) patients, respectively. Most patients (29.7%) who received ramucirumab as a second-line therapy were pretreated with lenvatinib. We found grade 3 or higher adverse events only in seven patients and no significant changes in the albumin-bilirubin score during ramucirumab treatment in the present cohort. The median progression-free survival of patients treated with ramucirumab was 2.7 months (95% confidence interval, 1.6–7.3).

**Conclusion:**

Although ramucirumab is used for various lines of treatment other than second-line immediately after sorafenib, its safety and effectiveness were not significantly different from the findings of the REACH-2 trial.

**Supplementary Information:**

The online version contains supplementary material available at 10.1186/s12876-023-02674-x.

## Introduction

Liver cancer has the sixth highest incidence among malignant tumors and the third highest in mortality rate worldwide with 781,631 deaths per year, and hepatocellular carcinoma (HCC) is the most common primary liver malignancy [1]. Monitoring high-risk populations, such as patients with cirrhosis due to hepatis B virus, hepatitis C virus (HCV), alcohol abuse, and nonalcoholic steatohepatitis, has increased the early detection of patients with HCC [2–4]. However, many patients are still diagnosed with advanced HCC in real-world practice.

Systemic therapies for advanced HCC have made significant progress in the past decade. Sorafenib, which was the first multikinase inhibitor for advanced HCC, improved overall survival (OS) compared with placebo in patients with advanced HCC in two randomized trials [5, 6]. Based on these results, sorafenib became the standard of care for systemic therapy of advanced HCC worldwide in the late 2000s. In the late 2010s, several drugs showed efficacy in randomized clinical trials in both first-line and second-line settings in patients with advanced HCC. Lenvatinib showed non-inferior survival to first-line treatment using sorafenib, and regorafenib, cabozantinib, and ramucirumab showed significantly better survival compared with placebo as a second-line treatment [7–10]. Furthermore, combination immunotherapies of atezolizumab plus bevacizumab and durvalumab plus tremelimumab were shown to significantly prolong OS compared with sorafenib treatment in patients with advanced HCC [11, 12]. Two combination immunotherapy regimens and five molecular target agents were shown to be effective against advanced HCC in global phase 3 randomized control trials and a wide variety of sequential treatments are being developed for clinical practice [5–12].

Ramucirumab is a human IgG monoclonal antibody that binds to the extracellular domain of VEGFR-2. It exerts its antitumor effects by inhibiting the interaction between VEGFR-2 and its ligand (mainly VEGF-A), thereby inhibiting endothelial cell proliferation and migration via downstream signaling [13–19]. The REACH trial, which was the first randomized, double-blind, placebo-controlled phase 3 clinical trials in a second-line setting after sorafenib, showed that ramucirumab failed to prolong OS compared with placebo [20]. A subgroup analysis of REACH trial revealed that ramucirumab was highly effective in patients with alpha-fetoprotein (AFP) levels > 400 ng/mL. Therefore, the REACH-2 trial compared ramucirumab and placebo in patients with advanced HCC after sorafenib in patients with AFP levels > 400 ng/mL [10]. The findings of the REACH-2 trial revealed that ramucirumab showed survival benefits compared with placebo and led to the use of ramucirumab as standard second-line treatment for advanced HCC. The biological mechanism that might explain the potential correlation between baseline AFP and the survival benefit of ramucirumab is uncertain. Robert M, et al. reported that AFP-high tumors showed higher enrichment of VEGF signaling and overexpression of VEGFB and PGF [21]. The overexpression of VEGFB and PGF ligands observed in AFP-high tumors might result in an enhanced activation of VEGFR1, as well as prevent VEGFA from binding VEGFR1. It means that the competition of VEGFA with the other ligands could favor its binding to VEGFR2. They conjectured a biological mechanism by which ramucirumab works against advanced HCC patients with AFP-high value as misbalance VEGFA signaling toward a preferential binding of VEGFR1.

In clinical practice, where multiple regimens are approved for advanced HCC, ramucirumab is used not only after sorafenib but also a post-treatment for various systemic therapies. However, little is known about the clinical outcomes of ramucirumab after other systemic therapies except sorafenib in patients with advanced HCC. Therefore, the present study aimed to evaluate the safety and effectiveness of various treatment lines of ramucirumab administration in real-world practice for patients with advanced HCC.

## Patients and methods

### Patients

In the present study, we included patients with advanced HCC who received ramucirumab in three institutions in Japan between June 18, 2019 (the date of ramucirumab approval in Japan), and March 30, 2021. In our clinical practice, per the Japanese guideline [22], we administered ramucirumab as second-or-later-line systemic therapy for advanced HCC patients for whom resection, local ablation, and transarterial chemoembolization (TACE) were not indicated as treatment options. We planned to exclude patients for whom ramucirumab was not used per the Japanese guideline from this study; however, not a single patient in this cohort met that criterion. Data were locked at the end of September 2021. This retrospective study was approved by the Research Ethics Committee of the Graduate School of Medicine, Chiba University (no. 3091). We had access to information that could identify individual patients during or after data collection although patients’ data were anonymized and de-identified prior to analysis.

### Ramucirumab treatment

Ramucirumab was injected intravenously at a dose of 8 mg/kg once every 2 weeks. The first dose was administered intravenously over 1 h and the administration time was reduced to 30 min from the second dose onward. In cases of unacceptable drug-related adverse events (AEs) as determined by physicians, ramucirumab was reduced or temporarily suspended until symptoms improved to grade 1 or 2 according to the manufacturer's guidelines.

Dynamic contrast-enhanced computed tomography or magnetic resonance imaging was performed at baseline and every 1–2 months after starting treatment to evaluate tumor response. Ramucirumab was continued until the physician determined clear progression of the disease using radiological imaging or the occurrence of AEs that prevented continuation of treatment.

### Clinical parameters

We retrospectively retrieved the medical records of all patients during the entire clinical course after administration of ramucirumab and collected data on the following clinical parameters: baseline demographic data of ramucirumab; date of radiological progression; reason for and date of discontinuation of ramucirumab; and date of death or last follow-up. Albumin-bilirubin (ALBI) scores were calculated at baseline and 2, 4, 6, 8, 10, and 12 weeks after ramucirumab administration to evaluate the transition of liver function. Radiological assessments were evaluated according to both Response Evaluation Criteria in Solid Tumours (RECIST) version 1.1 and modified RECIST (mRECIST) [23, 24]. AEs were evaluated according to the Common Terminology Criteria for Adverse Events version 5.0 [25].

### Statistical analysis

Kaplan–Meier plots of medians with 95% confidence intervals (CI) were used to estimate OS. The censoring date was defined as the date of the last follow-up. Progression-free survival (PFS) after ramucirumab was estimated using Kaplan–Meier plots of medians with 95% CI, with the date of progression defined according to RECIST and mRECIST and the censoring date defined as the date of last radiological assessment without progression. Repeated measures ANOVA was used to assess changes in ALBI score. *P*-values < 0.05 were considered statistically significant. All statistical analyses were conducted using Statistical Package for the Social Sciences version 25 statistical software (IBM, Armonk, NY, USA).

## Results

### Baseline characteristics

During the study period, a total of 37 consecutive patients received ramucirumab treatment for advanced HCC. Table 1 shows the baseline characteristics of the patients. The median age of the patients was 73 (47–86) years and most were male (31 patients, 83.8%). The most common etiology was HCV (37.8%), followed by hepatitis B virus (21.6%) and alcohol abuse (16.2%). The majority of the HCV patients (11 of 14) achieved sustained virological response. Most patients were Eastern Cooperative Oncology Group performance status grade 0 or 1 (97.3%) and Child–Pugh class A (81.0%) and 32.4% were diagnosed with liver cirrhosis. Thirty-two of the 37 patients had a history of pre-treatment before the administration of systemic therapies, including 10 (27.0%) with resection, 13 (35.1%) with local ablation, and 28 (75.7%) with TACE (duplicate cases available). The median AFP level was 1965.0 (409.0–9,3036.3) ng/mL. The median number of treatment cycles was 4 (1–24) and the median observation period was 8.0 (0.7–24.5) months.

**Table 1:** Baseline characteristics of 37 patients with hepatocellular carcinoma treated with ramucirumab

Demographics/characteristics	All patients (n = 37)
Sex, male	31 (83.8%)
Age, ≥ 73 years old	19 (51.4%)
HBs Ag-positive	8 (21.6%)
HBV DNA below detection limit due to nucleic acid	7 (18.9%)
HCV Ab-positive	14 (37.8%)
Achieved SVR	11 (29.7%)
Alcohol abuse	6 (16.2%)
Smoking history	23 (62.2%)
Body weight, < 60 kg	18 (48.6%)
Hypertension	24 (64.9%)
Diabetes mellitus	12 (32.4%)
Hyperlipidemia	11 (29.7%)
Liver cirrhosis	12 (32.4%)
ECOG-PS, ≤ 1	36 (97.3%)
Child–Pugh score	
5	17 (45.9%)
6	13 (35.1%)
≥ 7	7 (18.9%)
Number of intrahepatic lesions, ≥ 8	21 (56.8%)
Maximum size of intrahepatic lesions, > 50 mm	21 (56.8%)
Intrahepatic tumor occupation, ≥ 50%	3 (8.1%)
MVI	8 (21.6%)
EHM	18 (48.6%)
BCLC stage C	21 (56.8%)
Tumor differentiation	
Well differentiated	1 (2.7%)
Moderately differentiated	21 (56.8%)
Poorly differentiated	4 (10.8%)
Pre-treatment before administration of systemic therapy	32 (86.5%)
Resection	10 (27.0%)
Local ablation	13 (35.1%)
TACE	28 (75.7%)

HBs Ag, hepatitis B surface antigen; HCV Ab, hepatitis C virus antibody; SVR, sustained virologic response; ECOG, Eastern Cooperative Oncology Group; PS, performance status; MVI, macrovascular invasion; EHM, extrahepatic metastasis; BCLC, Barcelona clinic liver cancer; TACE, transarterial chemoembolization

**Fig. 1:**
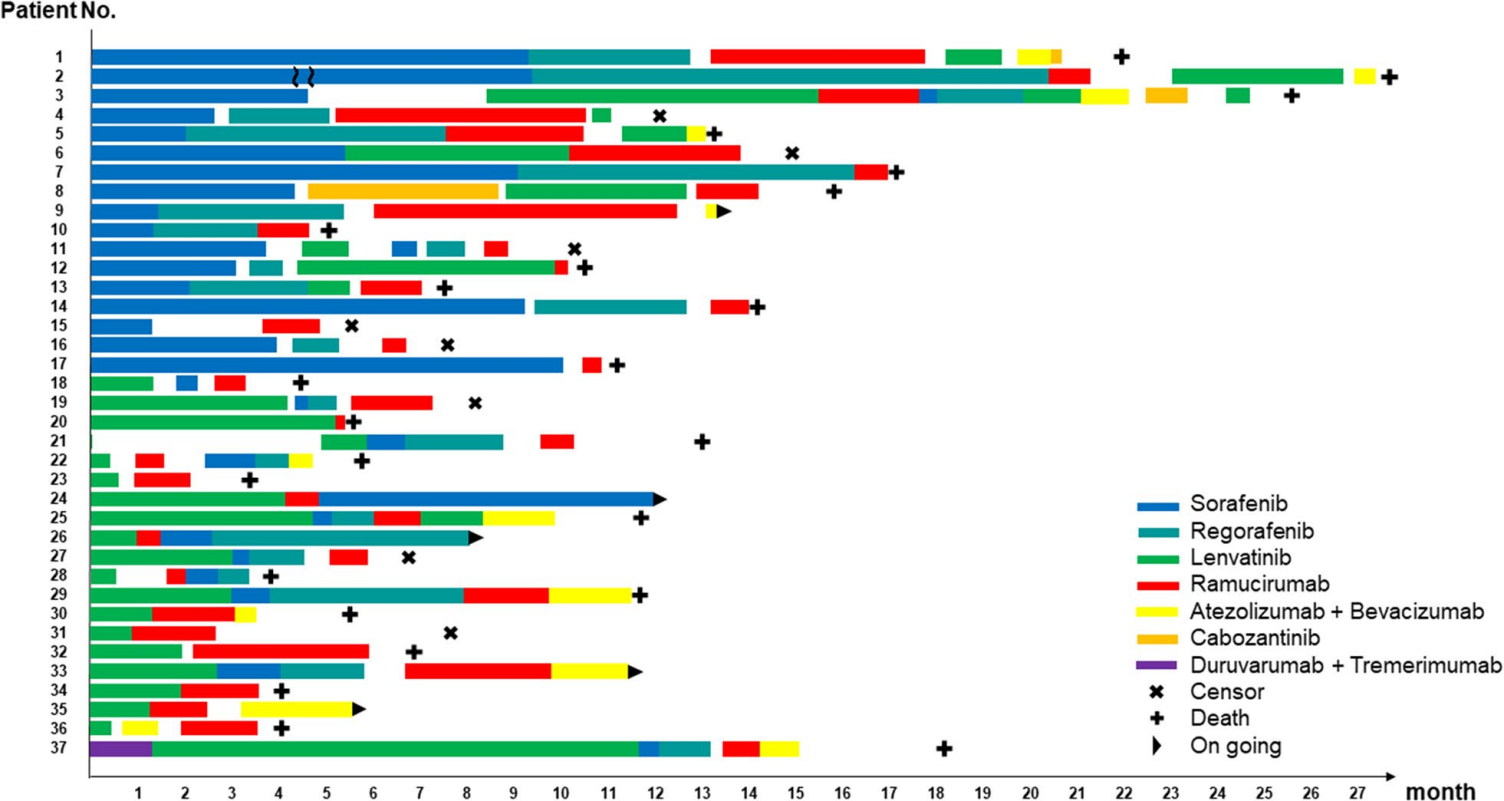
Treatment streams for all patients. Patients 1–17 are first-line sorafenib cases and 18–36 are first-line lenvatinib cases. Ramucirumab was administered as second, third, fourth, and fifth-line treatment in 13 (35.1%), 14 (37.8%), eight (21.6%), and two (5.4%) patients, respectively. The most common timing of ramucirumab administration was as second-line after lenvatinib in 11 (29.7%) patients. The longest duration of administration was 446 days (22 courses) and was third-line therapy after sorafenib and regorafenib. There was no consistent trend in the duration of ramucirumab treatment depending on the first-line drug and type of previous drug. In patient 2, the sorafenib administration period was 2,648 days; therefore, the bar indicating sorafenib administration period was shortened

Ramucirumab was administered as second, third, fourth, and fifth-line treatment in 13 (35.1%), 14 (37.8%), 8 (21.6%), and 2 (5.4%) patients, respectively (Fig. 1). Among 17 patients who received sorafenib as a first-line agent, 11 (64.7%) patients were administered regorafenib (11 patients). On the other hand, 11 out of 19 patients (57.9%) who were treated with lenvatinib as the first-line agent converted to ramucirumab for second-line treatment in the present cohort. We compared the backgrounds of patients who were administered ramucirumab as second-line and third-or-later-line therapy (Additional file 1: Table S1). There were significantly more patients with MVI in the second-line group than in the third-or-later-line group in the present study.

### Safety

**Fig. 2:**
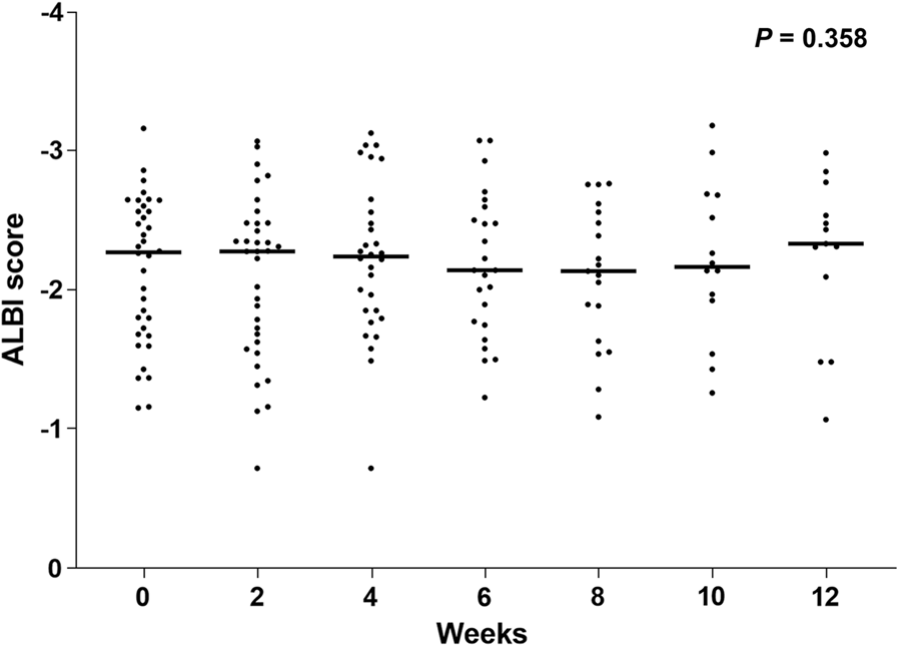
Changes in ALBI score during the disease course. Changes in ALBI scores at baseline and 2, 4, 6, 8, 10, and 12 weeks after ramucirumab administration. The median ALBI scores at baseline and 2, 4, 6, 8, 10, and 12 weeks were − 2.27, − 2.28, − 2.25, − 2.14, − 2.13, − 2.19, and − 2.38, respectively, with no significant change in ALBI scores during the study period (*P* = 0.358)

The AEs that occurred in our cohort during the observation period are shown in Table 2. The most frequent AEs for all grades were hypertension (n = 20; 54.0%), hypoalbuminemia (n = 15; 40.5%), proteinuria (n = 13; 35.1%), increased aspartate aminotransferase (n = 12; 32.4%), peripheral edema (n = 11; 29.7%), decreased platelet count (n = 9; 24.3%), anorexia (n = 8; 21.6%), and fatigue (n = 8; 21.6%). Grade ≥ 3 AEs were observed in seven patients (18.9%) and included hypertension (n = 2; 5.4%), fatigue (n = 2; 5.4%), anorexia (n = 2; 5.4%), duodenal ulcer (n = 1; 2.7%), proteinuria (n = 1; 2.7%), and hyperuricemia (n = 1; 2.7%). There were three (8.1%) patients who had to discontinue treatment solely because of AEs. Three patients (8.1%) had dose reductions during the study period, two of which were due to proteinuria. We evaluated changes in ALBI score from baseline to 12 weeks after ramucirumab administration (Fig. 2) and found no significant change in ALBI score within this period. We also compared patients who discontinued treatment due to AEs stratified by age (< 73 vs. ≥ 73), performance status (PS) (0 vs. ≥ 1), Child–Pugh class (A vs. B), and BCLC stage (A/B vs. C) (Table 3). In this study, the rate of treatment discontinuation due to AEs was significantly higher in Child–Pugh B patients.

**Table 2:** Adverse events during treatment with ramucirumab in patients with advanced hepatocellular carcinoma

Events	All patients (n = 37)	
	Any	Grade ≥ 3
Hypertension	20 (54.0%)	2 (5.4%)
Hypoalbuminemia	15 (40.5%)	0
Proteinuria	13 (35.1%)	1 (2.7%)
Increased aspartate aminotransferase	12 (32.4%)	0
Edema	11 (29.7%)	0
Decreased platelet count	9 (24.3%)	0
Anorexia	8 (21.6%)	2 (5.4%)
Fatigue	8 (21.6%)	2 (5.4%)
Increased alanine aminotransferase	7 (18.9%)	0
Bleeding	5 (13.5%)	1 (2.7%)
Elevated ammonia	4 (10.8%)	0
Diarrhea	3 (8.1%)	0
Increased serum amylase	2 (5.4%)	0
Anemia	2 (5.4%)	0
Blood bilirubin	2 (5.4%)	0
Fever	2 (5.4%)	0
Infusion reaction	1 (2.7%)	0
Weight loss	1 (2.7%)	0
Hyperuricemia	1 (2.7%)	1 (2.7%)

**Table 3:** Discontinuation rates due to adverse events in advanced hepatocellular carcinoma patients received ramucirumab

	All patient (n = 37)	*P* value
Any	4 (10.8%)	
Age		0.34
< 73 years (n = 18)	3 (8.1%)	
≥ 73 years (n = 19)	1 (2.7%)	
ECOG-PS		1
0 (n = 24)	0 (0%)	
≥ 1 (n = 13)	4 (2.7%)	
Child–Pugh class		0.016
A (n = 30)	3 (8.1%)	
B (n = 7)	1 (2.7%)	
BCLC stage		0.296
A or B (n = 16)	3 (8.1%)	
C (n = 21)	1 (2.7%)	

ECOG, eastern cooperative oncology group; PS, performance status; BCLC, Barcelona clinic liver cancer

### Effectiveness

The median OS from administration of ramucirumab in the present cohort was 10.3 months (95% CI, 5.2–14.6). The median PFS according to RECIST and mRECIST were 2.7 (95% CI, 1.6–7.3) and 2.7 (95% CI, 1.6–5.2) months, respectively. The best tumor responses according both RECSIT and mRECIST are shown in Additional file 1: Table S2. According to RECIST, partial response (PR), stable disease (SD), and progression disease (PD) were seen in one (2.7%), 18 (48.6%), and 14 patients (37.8%), respectively. The best tumor response based on mRECIST was seen in seven patients with PR (18.9%), 11 with SD (29.7%), and 13 with PD (35.1%). No patients achieved CR according to both RECIST and mRECIST.

According to RECIST, 18 out of 37 patients (48.6%) achieved PR or SD at imaging evaluation 28 days or later after administration (disease control ≥ 4 weeks). The OS in patients with disease control at ≥ 4 weeks was significantly longer than that in patients without disease control at ≥ 4 weeks (absence vs presence of disease control at ≥ 4 weeks: 5.2 months (95% CI 2.0–10.4) versus 14.1 months (95% CI 9.0–19.4; *P* = 0.043) (Additional file 2 Fig. S1).

**Fig. 3:**
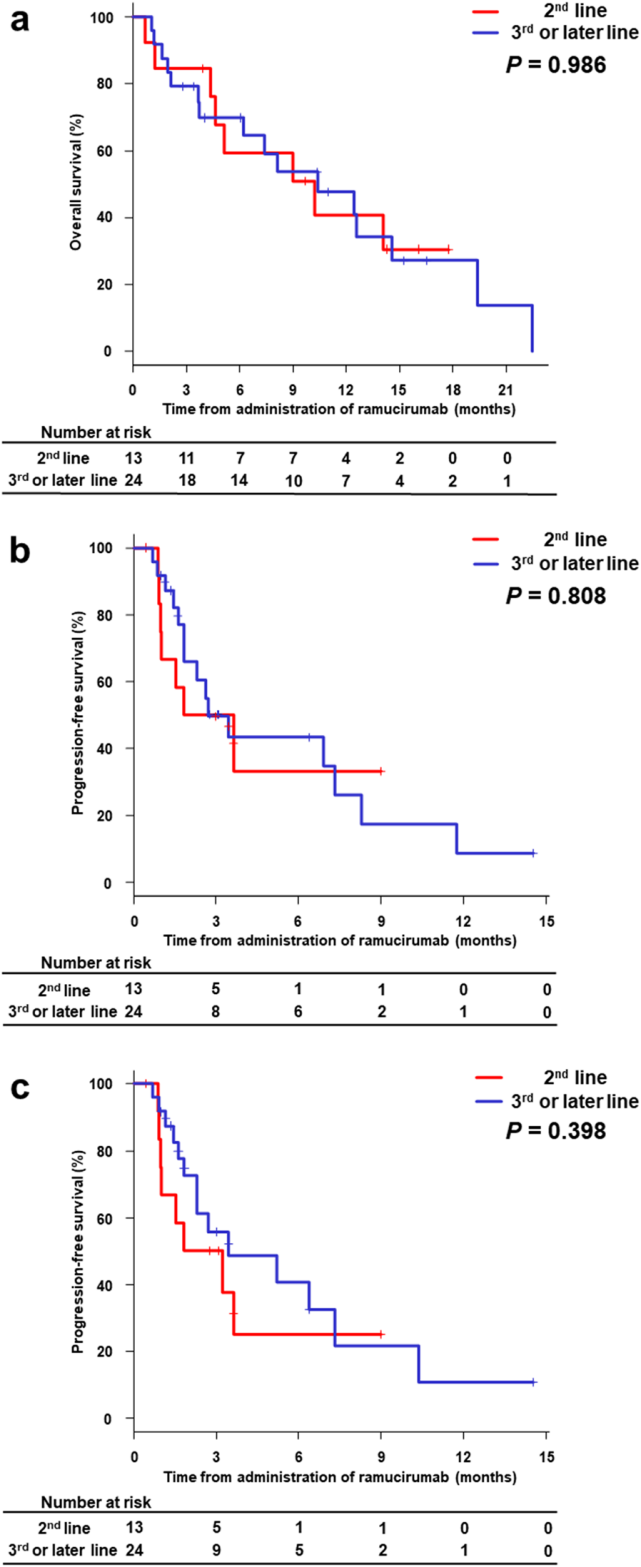
Kaplan–Meier curves for overall survival and progression-free survival. **a** The median overall survival for the second-line and late-line groups were 10.3 (95% CI 4.4–NA) and 10.4 (95% CI 3.7–14.6) months (*P* = 0.986), respectively. **b** The median progression-free survival for the second-line and late-line groups were 2.7 (95% CI 0.9–NA) and 2.7 (95% CI 1.6–7.3) months (*P* = 0.808), respectively, according to RECIST. (c) The median progression-free survival of the second-line and late-line groups were 1.8 (95% CI 0.9–NA) and 3.5 (95% CI 1.6–7.3) months (*P* = 0.398), respectively, according to mRECIST

We divided the patients into two groups according to treatment lines of ramucirumab: second-line (n = 13) and late-line (defined as third-line or later; n = 24). The median OS values of the second-line and late-line groups were 10.3 months (95% CI 4.4–not applicable (NA)) and 10.4 months (95% CI 3.7–14.6) (*P* = 0.986), respectively (Fig. 3A). The median PFS values of the second-line and late-line groups according to RECIST were 2.7 (95% CI 0.9–NA) and 2.7 (95% CI 1.6–7.3) months (*P* = 0.808), respectively (Fig. 3B). The median PFS values for the second-line and late-line groups according to mRECIST were 1.8 (95% CI 0.9–NA) and 3.5 (95% CI 1.6–7.3) months (*P* = 0.398), respectively (Fig. 3C). We analyzed OS and PFS by the presence or absence of tumor number > 7, AFP value > 1900 ng/mL (median value of this cohort), BCLC stage C, MVI, EHM, and Child–Pugh class B (Additional file 1: Table S3). In these results, Child–Pugh B patients had significantly shorter OS. There were no significant differences in the other parameters. We conducted multivariate analyses of OS and PFS using the Cox proportional hazard model with three variables (the treatment line, MVI, and Child–Pugh class) (Additional file 1: Table S4). These analyses showed that the treatment line did not contribute to both OS and PFS, and Child–Pugh B was an independent determinant of poor prognosis.

**Fig. 4:**
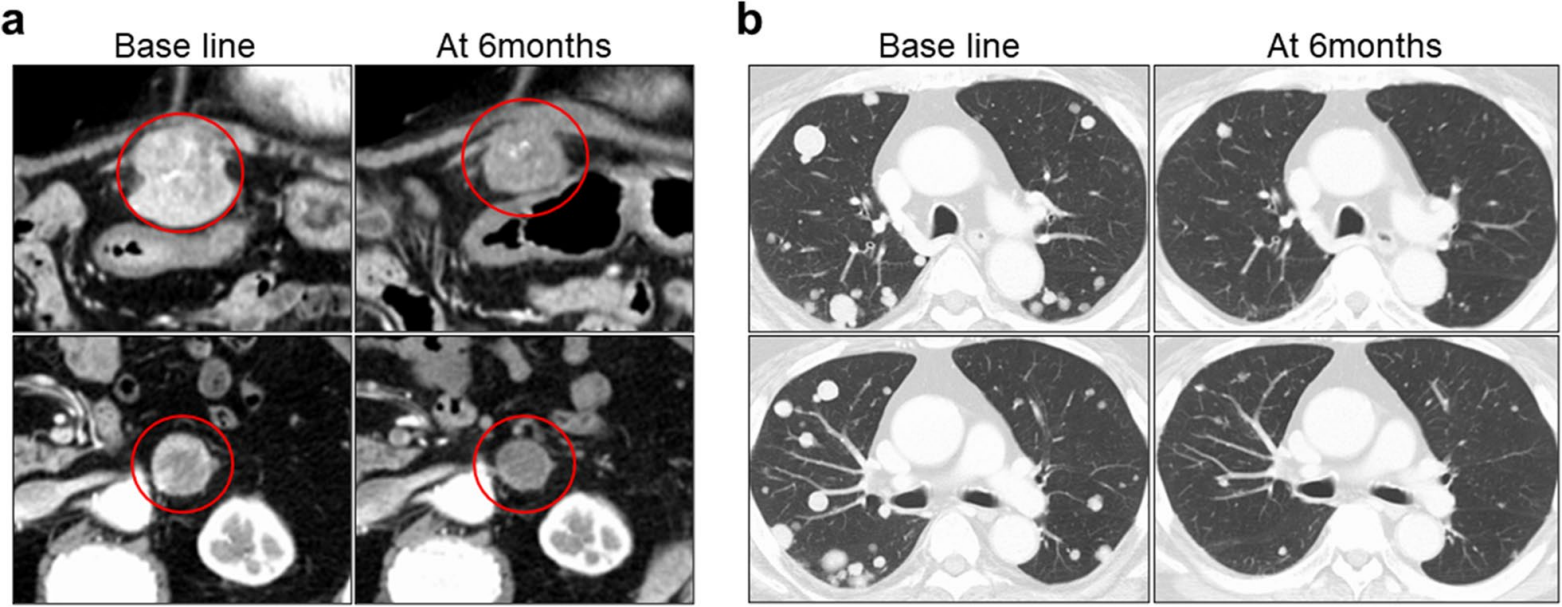
Imaging findings in patients who responded well to ramucirumab. Changes in imaging findings of **a** intrahepatic lesion and metastatic adrenal tumor and **b** metastatic lung tumors

Figure 4 shows the clinical course and indicates that ramucirumab was remarkably effective for advanced HCC patients in real-world practice. One patient was a 73-year-old man with cirrhosis caused by hepatitis B virus. He began ramucirumab as a third-line therapy after sorafenib and regorafenib. At the time of ramucirumab administration, we found one intrahepatic lesion (42.5-mm diameter) with portal vein invasion of the first branch, left adrenal metastasis, and multiple metastatic lung tumors on the baseline radiological assessment. Although the patient was evaluated as SD according to both RECIST and mRECIST at 2 months after administration, tumor shrinkages of both intrahepatic and extrahepatic lesions were gradually observed thereafter. PR was determined according to both RECIST and mRECIST at 6 months after starting ramucirumab treatment.

## Discussion

We examined the safety and effectiveness of various treatment lines of ramucirumab administration in patients with advanced HCC in real-world practice using a retrospective cohort in Japan. Ramucirumab was approved as second-line therapy after sorafenib due to the findings of the REACH-2 trial, which was designed when sorafenib was the only standard first-line systemic therapy for advanced HCC. However, in clinical practice, ramucirumab is currently administered in a wide variety of lines since multiple agents are available for advanced HCC. The results of the present study suggest the possible use of ramucirumab at diverse time points during the clinical course of advanced HCC.

In the present study, we focused on the safety of ramucirumab for advanced HCC patients in clinical practice. Although grade ≥ 3 AEs were observed in seven (18.9%) patients, only three (8.1%) patients required discontinuation of treatment due to AEs (one patient with tumor collapse, one patient with duodenal ulcer bleeding, and one patient with proteinuria). In recent clinical practice, regorafenib and cabozantinib are used as second-or-later-line treatments for advanced HCC patients as well as ramucirumab. We previously reported that regorafenib caused a high rate of grade 3 or higher AEs such as palmar-plantar eruthrodysesthesia (20.5%) and elevated serum aspartate aminotransferase (13.6%) in advanced HCC patients treated in clinical practice [26]. Similarly, other previous reports suggested that high rates of serious AEs and treatment discontinuation due to AEs occurred during regorafenib treatment in patients with advanced HCC [27, 28]. Francesco T et al. demonstrated that 42.7% of patients had grade 3 or higher AEs and 11.7% discontinued treatment due to AEs during cabozantinib treatment for advanced HCC patients [29]. Taken together with our results, ramucirumab would be the safest second-or-later-line treatment in patients with advanced HCC. Furthermore, as previously reported [30–33], analysis of the transition in ALBI score during the first 12 weeks after ramucirumab administration showed that deterioration of liver function was not observed in the present cohort. In contrast to sorafenib and lenvatinib, which have been reported to worsen liver function after initiation of treatment [34, 35], ramucirumab is considered to have an extremely low impact on liver function. In patients with advanced HCC, OS and PFS were correlated with duration of treatment using molecular target agents and impaired liver function is a known factor for preventing conversion to post-treatment [36–38]. Molecular target agents with a low risk of decreasing liver function may be a treatment option for patients with advanced HCC.

Also, the effectiveness of this study, the median OS was 10.3 months and the median PFS was 2.7 months using both RECIST and mRECIST. These data were comparable to the findings of the REACH-2 trial, which reported an OS of 8.5 months and PFS of 2.8 months. Interestingly, the median PFS for the second-line and late-line groups were similar using both RECIST and mRECIST (second-line vs late-line: RECIST, 2.7 vs 2.7 months; *P* = 0.945; mRECIST, 1.8 months vs 3.5 months; *P* = 0.409). In the preset study, first-line treatment was either sorafenib or lenvatinib, except in one patient who received duruvarumab plus tremerimumab as a clinical trial. Surprisingly, only 2 out of 37 patients (5.4%) were administered ramucirumab as second-line treatment after sorafenib and most patients (64.9%) started ramucirumab as late-line treatment. To date, ramucirumab as well as regorafenib and cabozantinib have been shown to be effective second-line treatment agents in phase 3 studies. However, the clinical trials of these three agents were all designed based the use of sorafenib as the first-line treatment of advanced HCC. Our results revealed that ramucirumab was used in a variety treatments line of for advanced HCC patients in real-world clinical practice.

In the present cohort, 11 out of 19 patients (57.9%) were administered ramucirumab as a second-line treatment after lenvatinib and showed a median PFS of 3.6 months. This finding is comparable to previous reports but remains unsatisfactory. Kuzuya et al. reported a median time to progression of 3.0 months in 12 patients with advanced HCC who were treated with ramucirumab after lenvatinib [30]. Similarly, Hiraoka et al. reported a median PFS of 2.0 months in patients with advanced HCC who were treated with ramucirumab after lenvatinib in [31]. At present, lenvatinib is the leading molecular target agent used for advanced HCC due to its high response rate. However, there is no evidence for second-line treatment after lenvatinib. We recently reported that use of sorafenib may be less effective after lenvatinib [39]. Taken together, our findings and the findings of previous studies indicate that ramucirumab may be a promising treatment option after lenvatinib in clinical practice.

In the present cohort, the median PFS was 7.3 months in patients who received ramucirumab after receiving sorafenib as first-line therapy. In addition, nine patients received ramucirumab after both sorafenib and regorafenib, including the patient shown in Fig. 4. We observed a favorable median PFS of 8.3 months in patients treated with sorafenib and regorafenib followed by ramucirumab. Although these results were derived from a small sample size, ramucirumab may a better treatment choice after sorafenib and regorafenib in patients with advanced HCC. We previously reported that sequential treatment of sorafenib and regorafenib was an effective treatment stream for advanced HCC in real-world practice [40]. Conversion to ramucirumab after sorafenib and regorafenib may lead to prolongation of prognosis in patients with advanced HCC.

The present study indicates the usefulness of ramucirumab for advanced HCC patients in clinical practice. OS was significantly longer in patients with confirmed disease control at ≥ 4 weeks after ramucirumab administration compared with those without disease control at ≥ 4 weeks. We previously reported that OS was prolonged in patients with confirmed SD at least 4 weeks after sorafenib administration [26]. For use of molecular target agents with less potential for tumor shrinking, such as sorafenib and ramucirumab, sustained control of tumor growth would be associated with survival in advanced HCC. It may be important to perform an initial radiological assessment 1 month after ramucirumab administration to determine whether patients would benefit from ramucirumab treatment. Early radiological evaluation during ramucirumab treatment may increase the likelihood of conversion to post-treatment when ramucirumab is refractory.

The present study had several limitations. First, in this study, we collected clinical data retrospectively. Although the results of our study showed that ramucirumab seemed to be safe in clinical practice, it is possible that we did not accurately collect data on all AEs that occurred during treatment. Second, the sample size of this study was small. It is often desirable to analyze factors contributing to safety and effectiveness from various perspectives using a variety of parameters. However, the sample size in this study was not large enough to perform such analyses. Third, our data dwelled on clinical outcomes of ramucirumab treatment when sorafenib or lenvatinib was used as the front-line treatment in patients with advanced HCC. Nowadays, combined immunotherapy is considered the first-line treatment for advanced HCC [11, 12]. The safety and effectiveness of ramucirumab after combined immunotherapy still needs to be validated. In conclusion, the results of the present study confirm the potential use of ramucirumab for various treatment lines of advanced HCC. Moreover, the lower impact of ramucirumab on liver function could be advantageous in strategies for treating patients with advanced HCC. Early radiological assessment is appropriate after the initiation of ramucirumab treatment in patients with advanced HCC.

## Supplementary Information

**Additional file 1. Table S1.** Comparing baseline characteristics of advanced hepatocellular carcinoma patients received ramucirumab in 2nd line and 3rd or later line. **Table S2.** Best response, objective response rate, and disease control rate during ramucirumab treatment. **Table S3.** Comparing overall survival and progression free survival by clinical parameters in advanced hepatocellular carcinoma patients received ramucirumab. **Table S4.** Multivariate analysis of OS, PFS during ramucirumab treatment; COX proportional hazards analysis.

**Additional file 2. Supplementary Figure 1:** Kaplan–Meier curves estimates of overall survival according to disease control status (SD ≥ 4 weeks). The median overall survival of patients with disease control ≥4 weeks and without disease control ≥ 4 weeks were 14.1 (95% CI, 9.0–19.4) and 5.2 (95% CI, 2.0–10.4) months (*P* = 0.043), respectively, according to RECIST and mRECIST.

## Data Availability

All data generated or analyzed during this study are included in this article and its online supplementary material files. Further inquiries can be directed to the corresponding author.
