# Supplementary material for: Use of ramucirumab for various treatment lines in real-world practice of patients with advanced hepatocellular carcinoma
Source: BMC Gastroenterol. 2023 Mar 11;23:70. doi: 10.1186/s12876-023-02674-x (PMC10007811; doi:10.1186/s12876-023-02674-x)
Supplement: Supplementary file 1 — Additional file 1. Table S1. Comparing baseline characteristics of advanced hepatocellular carcinoma patients received ramucirumab in 2nd line and 3rd or later line. Table S2. Best response, objective response rate, and disease control rate during ramucirumab treatment. Table S3. Comparing overall survival and progression free survival by clinical parameters in advanced hepatocellular carcinoma patients received ramucirumab. Table S4. Multivariate analysis of OS, PFS during ramucirumab treatment; COX proportional hazards analysis. [file 12876_2023_2674_MOESM1_ESM.docx]

**Supplementary Table 1.** Comparing baseline characteristics of advanced hepatocellular carcinoma patients received ramucirumab in 2^nd^ line and 3^rd^ or later line

|  | **2^nd^ line**  **(n = 13)** | **3^rd^ or later line**  **(n = 24)** | **P-value** |
| --- | --- | --- | --- |
| **Sex,** Male | 10 (76.9%) | 21 (87.5%) | 0.643 |
| **Age,** ≥73 | 8 (61.5%) | 11 (45.8%) | 0.495 |
| **Child-Pugh class,** B | 6 (46.2%) | 14 (58.3%) | 0.475 |
| **AFP,** ≥1900 | 5 (8.5%) | 14 (58.5%) | 0.475 |
| **BCLC,** C | 9 (69.2%) | 12 (50.0%) | 0.315 |
| **Tumor size, >**50mm | 8 (61.5%) | 13 (54.2%) | 0.739 |
| **Tumor number,** >7 | 7 (53.8%) | 14 (58.5%) | 1.000 |
| **MVI** | 6 (46.2%) | 2 (8.3%) | 0.013 |
| **EHM** | 6 (46.2%) | 12 (50.0%) | 1.000 |

AE, adverse event; BCLC, Barcelona clinic liver cancer; MVI, macrovascular invasion; EHM, extrahepatic metastasis

**Supplementary Table 2.** Best response, objective response rate, and disease control rate during ramucirumab treatment.

|  | **All patients (n = 37)** |
| --- | --- |
| **RECIST**  Complete response  Partial response  Stable disease  Progressive disease  Objective response rate  Disease control rate | 0  1 (2.7%)  18 (48.6%)  14 (37.8%)  1 (2.7%)  19 (51.4%) |
| **mRECIST**  Complete response  Partial response  Stable disease  Progressive disease  Objective response rate  Disease control rate | 0  7 (19.0%)  11 (29.7%)  13 (35.1%)  7 (19.0%)  17 (45.9%) |

RECIST, Response Evaluation Criteria in Solid Tumors; mRECIST, modified RECIST

**Supplementary Table 3.** Comparing overall survival and progression free survival by clinical parameters in advanced hepatocellular carcinoma patients received ramucirumab

|  | **OS** | | | **PFS (RECIST)** | | | **PFS (mRECIST)** | | |
| --- | --- | --- | --- | --- | --- | --- | --- | --- | --- |
|  | **Median (month)** | **95%CI** | **p-value** | **Median (month)** | **95%CI** | **p-value** | **Median (month)** | **95%CI** | **p-value** |
| **Tumor number >7**  Absent  Present | 14.1  6.2 | 7.43-NA  3.68-12.49 | 0.175 | 2.7  3.7 | 4.54-7.29  1.45-NA | 0.860 | 3.2  3.6 | 1.54-7.30  1.44-NA | 0.998 |
| **AFP >1900 ng/mL**  Absent  Present | 10.4  10.3 | 4.63-NA  1.97-NA | 0.874 | 3.6  2.7 | 1.45-8.18  1.84-NA | 0.860 | 3.5  3.4 | 1.45-10.35  1.84-6.37 | 0.617 |
| **Child-Pugh B**  Absent  Present | 12.5  1.64 | 7.43-19.41  0.69-NA | <0.001 | 2.7  3.5 | 1.61-7.30  0.69-NA | 0.803 | 3.2  3.5 | 1.61-6.37  0.69-NA | 0.955 |
| **BCLC C**  Absent  Present | 12.5  10.3 | 1.97-NA  4.40-19.42 | 0.742 | 6.9  2.6 | 1.84-NA  1.02-8.28 | 0.785 | 5.2  2.3 | 1.84-NA  1.02-3.50 | 0.659 |
| **MVI**  Absent  Present | 12.5  8.2 | 5.16-14.62  0.79-NA | 0.601 | 2.7  3.6 | 1.84-7.29  0.89-NA | 0.370 | 3.2  3.6 | 1.84-6.37  0.89-NA | 0.395 |
| **EHM**  Absent  Present | 9.0  12.6 | 3.75-14.09  3.68-19.42 | 0.360 | 6.9  1.8 | 1.84-NA  0.99-8.28 | 0.216 | 5.2  1.8 | 2.30-7.29  0.99-3.45 | 0.157 |

BCLC, Barcelona clinic liver cancer; MVI, macrovascular invasion; EHM, extrahepatic metastasis; OS, overall survival; PFS, progression free survival; RECIST, Response Evaluation Criteria in Solid Tumors; mRECIST, modified RECIST

**Supplementary Table 4.** Multivariate analysis of OS, PFS during ramucirumab treatment; COX proportional hazards analysis

|  | **OS** | | | **PFS (RECIST)** | | | **PFS (mRECIST)** | | |
| --- | --- | --- | --- | --- | --- | --- | --- | --- | --- |
| **Variables** | **Hazard ratio** | **95%CI** | **p-value** | **Hazard ratio** | **95%CI** | **p-value** | **Hazard ratio** | **95%CI** | **p-value** |
| **Treatment line**  2^nd^ line  3^rd^ or later line | Reference  1.63 | 0.284-2.226 | 0.370 | Reference  0.51 | 0.149-1.722 | 0.276 | Reference  0.43 | 0.127-1.486 | 0.184 |
| **Child-Pugh class**  A  B | Reference  4.76 | 1.61-14.110 | <0.01 | Reference  1.24 | 0.265-5.800 | 0.786 | Reference  1.10 | 0.235-5.145 | 0.904 |
| **MVI**  Absent  Present | Reference  0.80 | 0.561-4.735 | 0.663 | Reference  0.66 | 0.236-1.882 | 0.443 | Reference  0.48 | 0.173-1.354 | 0.167 |

OS, overall survival; PFS, progression free survival; RECIST, Response Evaluation Criteria in Solid Tumors; mRECIST, modified RECIST; MVI, macrovascular invasion
