# Supplementary figures and images for: Use of ramucirumab for various treatment lines in real-world practice of patients with advanced hepatocellular carcinoma
Source: BMC Gastroenterol. 2023 Mar 11;23:70. doi: 10.1186/s12876-023-02674-x (PMC10007811; doi:10.1186/s12876-023-02674-x)

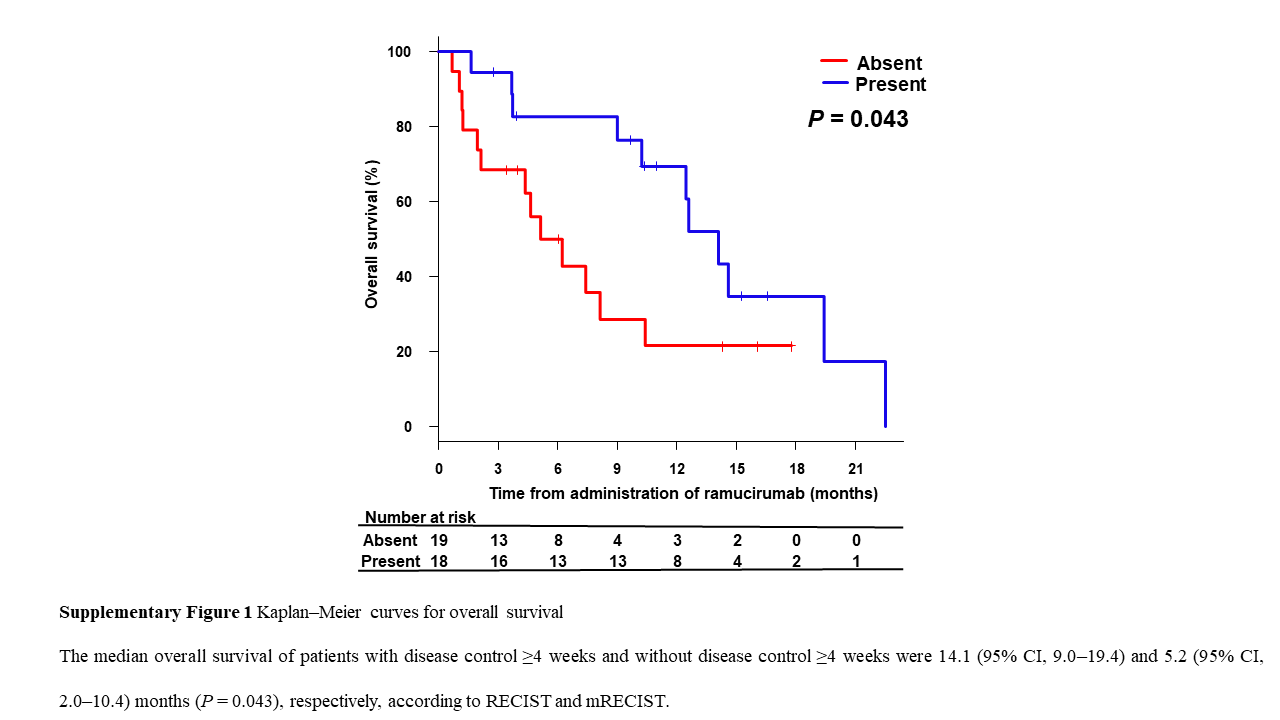

Supplement: Supplementary file 2 — Additional file 2. Supplementary Figure 1: Kaplan–Meier curves estimates of overall survival according to disease control status (SD ≥ 4 weeks). The median overall survival of patients with disease control ≥4 weeks and without disease control ≥ 4 weeks were 14.1 (95% CI, 9.0–19.4) and 5.2 (95% CI, 2.0–10.4) months (P = 0.043), respectively, according to RECIST and mRECIST. [file 12876_2023_2674_MOESM2_ESM.tif]
